# Association between sleep duration and cardiometabolic factors in adolescents

**DOI:** 10.1186/s12889-022-13119-7

**Published:** 2022-04-08

**Authors:** Susana Cararo Confortin, Liliana Yanet Gómez Aristizábal, Elma Izze da Silva Magalhães, Aline Rodrigues Barbosa, Cecilia Claudia Costa Ribeiro, Rosângela Fernandes Lucena Batista, Antônio Augusto Moura da Silva

**Affiliations:** 1 Federal University of Maranhão, Collective Health Post-Graduate Program, São Luís, Maranhão, Brazil; 2 School of Sports, Federal University of Santa Catarina, Florianópolis, Santa Catarina, Brazil

**Keywords:** Sleep, Glycemia, Arterial Pressure, Cholesterol HDL, Cholesterol LDL, Triglycerides

## Abstract

**Background:**

The sleep reduction can change healthy people's hemodynamic control and cardiovascular regulation through increased inflammatory response and altered endothelial function. The objective the study to analyze the association between sleep duration and cardiometabolic risk factors in adolescents in the birth cohort of São Luís (1997/98).

**Methods:**

This is a cross-sectional study with adolescents participating in the birth cohort of São Luís (1997/98). Sleep duration was evaluated using accelerometer data (Actigraph wGT3X-BT). Glycemia, systolic blood pressure (SBP), diastolic blood pressure (DBP), total cholesterol, low-density lipoprotein cholesterol (LDL-c), high-density lipoprotein cholesterol (HDL-c) and triglycerides were considered cardiometabolic factors. The Directed Acyclic Graph (DAG) was used to identify the minimum set of adjustment for confounding.

**Results:**

Out of 1,268 adolescents, 50.3% of them were male. The prevalence of sleep duration of less than 6 h per day was 31.1%. The mean glycemia value was 91.8 mg/dL (± 15.9), DBP was 71.3 mmHg (± 7.5), SBP was 114.9 mmHg (± 12.3), HDL was 48.5 mg/dL (± 11.6), LDL was 89.0 mg/dL (± 25.7), the total cholesterol was 156.0 mg/dL (± 31.1), and triglycerides was 93.6 mg/dL (± 47.2). The crude analysis showed an association between sleep duration and SBP and LDL-c. In the adjusted analysis, the associations did not remain.

**Conclusion:**

Our study showed no association between sleep duration and cardiometabolic outcomes in adolescents.

**Supplementary Information:**

The online version contains supplementary material available at 10.1186/s12889-022-13119-7.

## Background

Sleep is considered an important psychological and biochemical factor, which contributes to optimal health and vitality [1]. According to the literature, the variability of sleep patterns, especially sleep duration, can disrupt circadian cycles of biological processes, resulting in metabolic and endocrine dysfunction, which increase cardiovascular risk [2–4].

In contrast to those observed during wakefulness, the hemodynamic changes found during the sleep period are one of the possible explanations of the relationship between sleep and cardiovascular risk [5, 6]. Thus, sleep reduction can change healthy people's hemodynamic control and cardiovascular regulation through increased inflammatory response and altered endothelial function [7, 8].

The National Sleep Foundation (NSF), in consensus with a multidisciplinary panel of experts, recommends that individuals between 18 and 25 years old sleep 7 to 9 h per night. This sleep time is considered adequate for health and well-being, emphasizing that some individuals may sleep more or less than this time without adverse effects. However, people with sleep durations too far outside the recommended range may either be involved in sleep deprivation or have health problems [9].

The short duration of sleep has become increasingly widespread due to the effect it has on health and quality of life. Current evidence indicates that inadequate sleep duration may play a role in cardiometabolic risk for children and adolescents and with consequences later age [10, 11]. There is evidence of an association between inadequate sleep and abdominal adiposity [12], decreased insulin sensitivity [13], and high blood pressure [14]. However, evidence of potential associations between sleep and lipids, and inflammatory markers, are less convincing [10–13, 15].

Although national [13, 15, 16] and international studies [6, 17, 18] have investigated cardiometabolic risk factors, researches involving adolescents are still scarce. The adverse effects of inadequate sleep duration on physiological functions and cardiovascular risk factors that may underlie the association with increased morbidity and mortality are not fully understood [19].

Thus, understanding the possible adverse effects of sleep duration on these risk factors can help implement interventions aimed at improving adolescents’ sleep habits and, consequently, reduce the emergence of cardiometabolic risk factors throughout life. Therefore, this study aims to analyze the association between sleep duration and cardiometabolic factors in adolescents in the birth cohort of São Luís (1997/1998), in the northeast of Brazil (1997/98).

## Methods

This cross-sectional study is part of the birth cohort study (1997/98) in São Luís, State of Maranhão. The methods related to the sample planning of the cohort baseline and follow-ups were previously published and will be explained briefly [20].

The perinatal study of the São Luís cohort was initiated in 10 public and private hospitals in the city, from March 1997 to February 1998, entitled “Determinants throughout the life cycle of obesity, precursors of chronic diseases, human capital and mental health”. After the exclusion of stillbirths, the final sample consisted of 2,493 births. We used data from the third phase of the cohort conducted between January and November 2016, in which participants were already 18/19 years of age. In total, 687 individuals were located and agreed to participate in the study. In this follow-up, a retrospective component was included (with application of a fundamental part of the perinatal questionnaire to mothers of adolescents), conducted with 1,828 adolescents born in São Luís in 1997 and who were initially not drawn to participate in the cohort in the previous phases of the study were included totaling 2,515 adolescents. The new members were selected by random sampling from the System of Information about Liveborns restricting for children born in 1997. Adolescents identified in schools and universities and by means of the social network were also included [20, 21]. Information from 1,538 individuals who used the accelerometer was collected. Of the 1,538 individuals, 179 were excluded due to invalid data and 35 excluded due to some error or defect (calibration error greater than 0.02, patterns not compatible with human movement, incomplete 24-h cycle, and presented quality problems after visual inspection of the plots) (Figure S1).

Furthermore, according to Spearman Brown's formula, 56 adolescents were excluded because they had incomplete data (use of fewer than four days). Thus, the final sample of the São Luís birth cohort in this study was composed of 1,268 adolescents.

### Cardiometabolic risk factors

The following cardiometabolic risk factors were considered: blood pressure, glycemia, total cholesterol, triglycerides, low-density lipoprotein cholesterol (LDL-c) and high-density lipoprotein cholesterol (HDL-c).

#### Arterial Pressure

Both systolic blood pressure (SBP) and diastolic blood pressure (DBP) were measured with the Omron HEM 742INT device (Omron, São Paulo, Brazil), three times in each arm, after five minutes at rest [21]. The mean of the three measurements was used.

#### Glycemia

Without fasting, the glycemia was determined by the enzymatic colorimetric method automated by the Cobas equipment® (Module cobas c 501) from Roche®. The variable was used continuously (mg/dL).

#### Total cholesterol, triglycerides, low-density lipoprotein cholesterol (LDL-c) and high-density lipoprotein cholesterol (HDL-c)

Total cholesterol, triglycerides, LDL-c, HDL-c were dosed the enzymatic colorimetric method automated by the Cobas equipment® (Module cobas c 501), Roche®.

Blood collection was performed through venipuncture, strictly following the recommendations of good practices in clinical analysis laboratories (http://www.ufma.br/portalUFMA/arquivo/3c85c88c4fc6e33.pdf), and identification is performed at the front of the individuals with barcode labels with the patient's name and exams to be performed, as well as the material is all disposable. Upon arrival at the laboratory, the samples are checked and sent from the technical sectors to carry out the necessary analyzes. In addition, the laboratory follows the requirements of Anvisa (*Agência Nacional de Vigilância Sanitária*—National Health Surveillance Agency), provided for in the Resolution of the Collegiate Board (RDC—*Resolução da Diretoria Colegiadas*) 302/2005, for quality control, safety and efficiency in exams. For laboratory tests, fasting was not required. Continuous values were considered for each factor.

### Sleep Duration (independent variable)

Sleep duration was evaluated using an accelerometer (GT3X and GTX3 + models) of *Actigraf®,* collected with a frequency of 60 Hz and epochs of 5 s. Calibration of all accelerometers and the training of those involved in the collection was performed. The survey supervisors explained the usage procedures and invited the individuals to use the accelerometer for 7 continuous days. The instrument should be used 24 h a day, except in bathing and water activities. The crude data were extracted using the Software ActiLife version 6.12, resulting in a spreadsheet (.csv) for each adolescent. Data processing after extraction was performed using the R statistical package (GGIR package version 6.12). The non-human movement filtering, the validation of time of use, calibration, the data quality graphs, and others were performed. Of the 1,538 individuals, 179 were excluded due to invalid data and 35 excluded due to some error or defect (calibration error greater than 0.02, patterns not compatible with human movement, incomplete 24-h cycle, and presented quality problems after visual inspection of the plots). Moreover, we excluded 56 due to insufficient data (use of less than 4 days). Spearman Brown’s formula was applied to test the reliability for the minimum days of use of the accelerometer for sleep [22], parameters, being defined at least 4 nights, with a value of 0.52, being the best result considering the loss of many observations. Sleep duration was identified based on the algorithm proposed by Van Hess et al. [23]. It was categorized into 6 h or more and less than 6 h of sleep, depending on the population's average, since less than 20 participants had 8 h or more of sleep.

### Complementary variables

The variables age (in continuous years), gender (male and female), skin color (white, black and mixed-race), adolescent's years of study, socioeconomic classification assessed by Economic Classification Brazil 2016 (Classificação Econômica Brasil – CEB) criterion A/B (B1 + B2), C(C1 + C2), D/E, class A/B being the richest and schooled and D/E the poorest and least educated [24]. Current work and smoking (yes/no), and alcohol consumption (yes/no) were also evaluated [25]. The consumption of in natura or minimally processed foods and ultra-processed foods [26] (%kcal) was assessed using a validated food frequency questionnaire (FFQ) for Brazilian adolescents [27], categorized into tertiles. Physical activity was assessed by accelerometer, in minutes/day of moderate and vigorous physical activity [28], being categorized into tertile (1st tertile: < 15 min 50 s; 2nd tertile: ≥ 15 min 50 s and < 36 min 15 s; 3rd tertile: ≥ 36 min 15 s). Screen time (non-use < 2 hs; ≥ 2 hs to ≤ 5 hs; > 5 hs) was considered exposure to electronic devices, such as mobile phones, tablets, computers, video games, and television during weekdays, whereas weekends were disregarded in the evaluation. Body mass index (BMI) was estimated from the ratio of body mass, in kilograms, by height, in meters, squared (BMI kg/m^2^ = body mass/height^2^). To evaluate the major depressive episode or depression (yes/no), the M.I.N.I. Questionnaire was used. (Mini International Neuropsychiatric Interview – Brazilian version 5.0.0—DSM IV) [24].

### Data analysis

Descriptive analyses were performed with estimates of absolute frequencies and percentages. The mean outcomes between the groups were compared using the Student’s *t*-test or Wilcoxon Test and one-way ANOVA or Kruskal–Wallis Test.

**Fig. 1:**
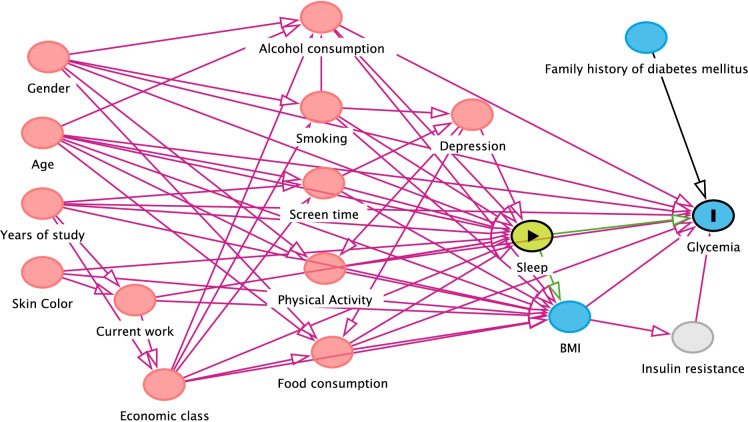
Acyclic graph directed to the association between sleep and glycemia

**Fig. 2:**
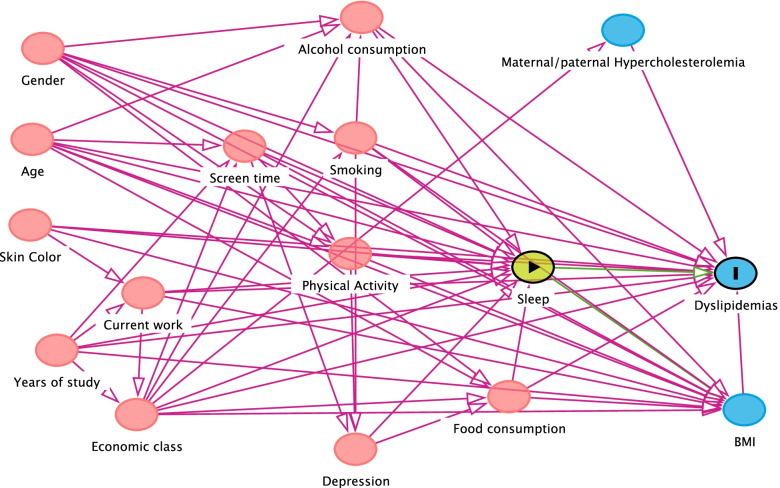
Acyclic graph directed to the association between sleep and dyslipidemias

**Fig. 3:**
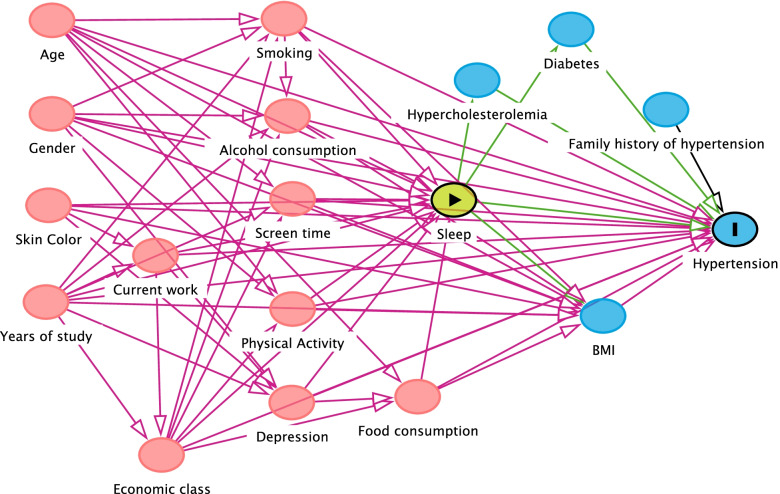
Acyclic graph directed to the association between sleep and blood pressure

Regarding the covariates of the study, a directed acyclic graph (DAG) was built from the literature review on the study theme, which shows the complex relationship between the different risk factors (Figs. 1, 2, and 3). DAG is a visual and qualitative tool for selecting confounding variables identified from a theoretical model. The arrowheads inform a path between two variables, and it is possible, through pre-established rules, to identify a minimum set of variables for adjustment. After application of DAG’s rules, the minimum adjustment set of variables for analysis of association between sleep duration and cardiometabolic outcomes (blood pressure, dyslipidemia and glycemia) were: For the associations between sleep and diabetes, the following variables were selected: age, gender, adolescent years of study, skin color, economic class, current work, alcohol consumption, smoking, physical activity, screen time, food consumption. For sleep and blood pressure, in turn, the variables selected were adjusted for: age, gender, adolescent years of study, skin color, economic class, current work, alcohol consumption, smoking, physical activity, screen time, food consumption, depression. In the association between sleep and dyslipidemias, the adjustment variables were: age, gender, adolescent years of study, skin color, economic class, current work, alcohol consumption, smoking, physical activity, screen time, food consumption.

To analyze the relationship between sleep duration and cardiometabolic outcomes, a weighted by propensity score was used from the Inverse Probability of Treatment Weighting (IPTW). This study used the propensity score as a way to correct the imbalance in the distribution of predictors (covariate) that occur in observational studies, thus making the groups comparable and avoiding biased estimates of the effect of sleep duration and cardiometabolic outcomes.

The propensity score calculation was performed using the linear regression. The application of linear regression allowed the estimation of the selection probability of adolescents, conditional on the values of covariates identified in the DAG (Figs. 1, 2 and 3).

Checking the common support area and balancing the groups verified adequate propensity score estimation. A balance in the distribution of observed covariates was achieved, obtaining standardized differences between means and proportions less than 0.1 and for variances between 0.18–1.2. The effect of of sleep duration on cardiometabolic outcomes was calculated using the beta coefficient with 95% confidence intervals (95% CI) for each cardiometabolic outcome considered in the analysis. No interaction was observed between sleep duration and gender concerning glycemia, blood pressure, and dyslipidemia outcomes evaluated by the gender/sleep duration ratio. Therefore, the sample was not stratified by gender.

Statistical analysis was conducted in Stata 14.0 (Stata Corporation, College Station, Texas, USA).

### Ethical aspects

The project referring to the birth cohort of São Luís (1997/98) was approved by the Research Ethics Committee of the University Hospital—Federal University of Maranhão (UFMA), under Opinion no. 1.302.489. In all phases of the cohort, the Informed Consent Form was signed by the individual or guardian. All projects meet the criteria in Resolution (466/2012) of the National Health Council and its complementary regulations.

## Results

Studies have assessed the distribution of participants according to the use or not of the accelerometer, with differences between the categories of age (*p* ≤ 0.001), gender (*p* ≤ 0.001), schooling (*p* ≤ 0.001), skin color (*p* = 0.016), economic class (*p* ≤ 0.001), leisure-time physical activity (*p* ≤ 0.001), drug use (*p* = 0.008). Most participants who used the accelerometer were 18 years old (86.8%), had lower schooling (71.6%), were male (50.45%), the poorest (Class C) (49.5%), and the most physically active (42.9%) [29].

The mean glycemia value was 91.8 mg/dL (± 15.9), diastolic blood pressure was 71.3 mmHg (± 7.5), and systolic blood pressure was 114.9 mmHg (± 12.3), HDL was 48.5 mg/dL (± 11.6), LDL was 89.0 mg/dL (± 25.7), total cholesterol was 156.0 mg/dL (± 31.1), triglycerides was 93.6 mg/dL (± 47.2) and mean sleep time was 6 (± 1.0) hours per day. The prevalence of sleep less than 6 h per day was 31.1% (Table 1).

**Table 1:** Characterization of the sample and mean and standard deviation of outcomes according to demographic, socioeconomic, lifestyle, and sleep duration characteristics in adolescents (*n* = 1,268). São Luís, Maranhão, Brazil, 2016/2017

**Variable**		**Glycemia** **(mg/dL)**	**DBP** **(mmHg)**	**SBP** **(mmHg)**	**HDL-c** **(mg/dL)**	**LDL-c** **(mg/dL)**	**Cholesterol** **total (mg/dL)**	**Triglycerides** **(mg/dL)**
	***n* (%)**	**Mean (SD)**	**Mean (SD)**	**Mean (SD)**	**Mean (SD)**	**Mean (SD)**	**Mean (SD)**	**Mean (SD)**
**Total**	1,268	91.8(15.9)	71.3 (7.5)	114.9 (12.3)	48.5(11.6)	89.0 (25.7)	156.0 (31.1)	93.6 (47.2)
**Age (*n *= 1,268)**		*p* = 0.327**	*p* = 0.839*	*p* = 0.011**	*p* = 0.010**	*p* = 0.171*	*p* = 0.038*	*p* = 0.986*
18 years	1105(87.1)	91.9 (16.1)	71.4 (7.5)	115.2 (12.4)	48.2 (11.5)	88.5(25.2)	155.2(30.4)	93.3 (46.2)
19 years	163 (12.9)	90.6 (1.2)	70.5 (7.2)	112.0 (11.6)	50.7 (11.9)	92.7 (28.7)	161.8 (35.1)	95.4 (53.6)
**Gender (*n* = 1,268)**		< 0.001*	*p* = 0.030**	< 0.001*	< 0.001*	*p* < 0.001**	*p* < 0.001**	*p* < 0.001**
Male	638 (50.3)	92.7 (17.0)	72.2 (7.7)	121.4 (10.9)	45.0 (10.2)	85.9 (24.9)	150.6 (29.2)	99.7 (49.2)
Female	630 (49.7)	90.8 (14.7)	70.3 (7.2)	108.2 (9.9)	52.1(11.8)	92.2 (26.1)	161.7(31.9)	87.2(44.2)
**Adolescent years of study (*n* = 1,261)**		*p* = 0.090*	*p* = 0.042**	*p* = 0.606**	*p* = 0.207**	*p* = 0.408**	*p* = 0.094**	*p* = 0.830
No schooling at 11 years	1,202 (95.3)	92.0 (16.0)	71.2 (7.5)	114.9 (12.4)	48.4 (11.5)	88.9(25.5)	155.6 (31.1)	93.4 (47.3)
12 or more	59(4.7)	87.6(11.3)	71.7(7.3)	114.1 (11.6)	50.3 (12.5)	91.8 (29.7)	162.7(31.1)	94.8 (44.4)
**Skin Color (*n* = 1,261)**		*p* = 0.107****	*p* = 0.175****	*p* = 0.213****	*p* = 0.034***	*p* = 0.107***	*p* = 0.177*	*p* = 0.137****
White	235 (18.6)	92.5 (15.2)	70.6 (7.0)	114.5 (11.6)	48.2(11.9)	89.9(28.0)	156.0(33.4)	93.1 (46.6)
Black	202 (16.0)	89.6 (14.8)	71.3 (7.2)	114.9 (13.1)	50.8(12.8)	89.5(14.9)	159.2(31.1)	92.6 (51.7)
Mixed race	824 (65.4)	92.1 (16.4)	71.4 (7.7)	114.9 (12.3)	48.0 (11.1)	88.7 (25.2)	155.3 (30.3)	93.9 (46.4)
**Economic class (*n* = 1,110)**		*p* = 0.367****	*p* = 0.536****	*p* = 0.703*****	*p* = 0.483****	*p* = 0.150***	*p* = 0.087****	*p* = 0.069****
A/B	308 (27.8)	90.4 (14.5)	70.8 (7.1)	115.0 (12.2)	49.0(11.2)	89.8(26.5)	157.5(31.7)	93.4 (45.3)
C	552 (49.6)	91.6 (14.1)	71.3 (7.5)	115.1 (12.5)	48.7 (11.8)	88.0 (24.5)	154.6 (29.7)	92.3 (46.7)
D/E	251 (22.6)	92.6 (15.2)	71.2 (7.3)	113.1(12.0)	48.5 (12.0)	91.1(26.8)	158.7(33.4)	95.0 (51.7)
**Current works (*n* = 1,268)**		*p* = 0.568**	*p* = 0.242**	*p* = 0.727**	*p* = 0.909**	*p* = 0.631**	*p* = 0.811**	*p* = 0.584**
No	1,066 (84.1)	91.9 (16.2)	71.4 (7.6)	11.8 (12.4)	48.5(11.5)	88.9(25.7)	155.9(31.0)	93.2 (47.1)
Yes	2,020 (15.9)	91.2 (14.6)	70.7 (7.2)	115.4(11.9)	48.6(11.8)	89.8(25.5)	156.5 (31.2)	95.3(47.8)
**Alcohol consumption (*n* = 1,257)**		*p* = 0.742*	*p* = 0.906**	*p* = 0.347**	*p* = 0.705**	*p* = 0.304**	*p* = 0.896**	*p* = 0.179**
No	744 (59.1)	91.7 (14.8)	71.1 (7.5)	114.6 (12.1)	48.4(11.9)	89.7(25.6)	155.9(31.5)	91.3(45.4)
Yes	513 (40.9)	91.8 (17.1)	71.5 (7.6)	115.3 (12.6)	48.7(1.1)	88.2(25.6)	156.2(30.3)	96.4 (48.0)
**Smoking (*n* = 1,268)**		*p* = 0.189**	*p* = 0.704**	*p* = 0.528*	*p* = 0.127**	*p* = 0.876**	*p* = 0.512**	*p* = 0.044**
No	1,228 (96.9)	91.7 (14.6)	71.2 (7.5)	114.8 (12.2)	48.6 (11.5)	89.0 (25.6)	155.9 (30.9)	93.1 (47.3)
Yes	40 (3.1)	94.3 (38.7)	72.0 (8.3)	116.9 (15.1)	45.7(11.7)	89.6(28.9)	159.2 (34.7)	108.3 (42.8)
**Tertile physical activity (*n* = 1,268)**		*p* = 0.148***	*p *= 0.113****	*p* = 0.297****	*p* = 0.710****	*p* = 0.522****	*p* = 0.009***	*p* = 0.585****
1^st^ tertile (< 15 min 50 s)	426 (33.6)	91.0 (15.9)	71.1 (7.2)	113.0(11.7)	48.6(11.6)	90.8(25.5)	157.7(30.5)	92.9 (46.6)
2^nd^ tertile (≥ 15 min 50 s and < 36 min 15 s)	427 (33.7)	92.1 (17.7)	71.1 (7.4)	114.5 (12.5)	49.3(11.7)	89.9(26.4)	157.8(33.2)	93.1 (48.6)
3^rd^ tertile (≥ 36 min 15 s)	415 (32.7)	92.3 (13.9)	71.5 (9.9)	117.2 (12.5)	47.5(11.3)	86.3(25.0)	152.4(29.2)	94.7 (46.5)
**BMI (*n* = 1,253)**		*p* = 0.664***	*p* = 0.120****	*p* = 0.077****	< 0.001***	*p* < 0.001***	< 0.001***	*p* < 0.001***
Adequate	1,011 (80.7)	91.9 (16.6)	70.5 (7.3)	113.3 (11.6)	49.2(11.3)	87.2 (24.2)	153.8 (28.6)	87.8 (40.9)
Overweight	190 (15.2)	91.7 (12.3)	73.4 (7.0)	120.7(12.6)	46.2(12.6)	94.3(27.7)	162.1 (35.3)	110.6 (56.6)
Obesity	52 (4.1)	92.3 (14.3)	78.3 (8.7)	124.4 (14.0)	40.6(9.5)	103.0(35.0)	170.2(39.6)	132.3 (62.9)
**Food ultra-processed (tertile) (*n* = 1,254)**		*p* = 0.917***	*p* = 0.424****	*p* = 0.404****	*p* = 0.588****	*p* = 0.781****	*p* = 0.496****	*p* = 0.070****
1^st^ tertile	426 (34.0)	91.9 (16.7)	70.9 (7.6)	115.7 (12.5)	47.9(10.9)	88.5 (25.3)	154.7 (30.1)	93.4 (44.5)
2^nd^ tertile	396 (31.6)	92.0 (14.0)	71.4 (7.7)	115.3 (12.6)	48.8 (12.4)	88.2 (26.2)	156.1 (31.9)	94.0 (49.9)
3^rd^ tertile	432 (34.4)	16.8 (16.8)	71.4(7.2)	16.8 (16.8)	48.7 (11.4)	90.1(25.5)	157.2 (31.3)	93.3 (47.7)
**Food in natura or minimaly processed (tertile) (*n* = 1,254)**		*p* = 0.980***	*p *= 0.252****	*p* = 0.146***	*p* = 0.172****	*p* = 0.753****	*p* = 0.510****	*p* = 0.944***
1^st^ tertile	439 (35.0)	91.4 (12.9)	71.4(7.2)	113.9 (11.7)	48.9 (11.5)	90.4 (26.1)	157.8 (32.0)	94.4 (49.6)
2^nd^ tertile	384 (30.6)	92.6 (18.4)	71.2 (7.4)	114.8 (12.2)	48.6 (12.15)	87.6 (25.2)	155.2 (30.6)	93.8 (48.2)
3^rd^ tertile	431 (34.4)	91.6 (16.5)	71.1 (7.8)	116.1 (12.9)	48.0 (11.0)	88.9 (25.6)	154.8 (30.4)	92.5 (44.1)
**Depression**		*p* = 0.027*	*p* = 0.152*	*p* < 0.001*	*p* = 0.113**	*p* = 0.858**	*p* = 0.307**	*p* = 0.514**
No	1.128(88.9)	92.2(16.3)	71.4(7.6)	115.4(12.5)	48.3(11.5)	89.0(25.5)	155.7(30.9)	93.3(47.3)
Yes	140(11.1)	88.7(11.6)	70.2(6.6)	110.4(9.7)	50,0(12.1)	89.4(26.8)	158.6(32.0)	96.1(46.3)
**Sleep duration (*n* = 1,268)**		*p* = 0.857*	*p* = 0.202**	0.001**	*p* = 0.145**	***p*** = 0.036**	*p* = 0.098**	*p* = 0.980*
≥ 6 h	874 (68.9)	91.5 (14.2)	71.1 (7.5)	114.0 (12.5)	48.8 (11.4)	90.1 (26.2)	157.0(31.4)	92.6 (45.1)
< 6 h	394 (31.1)	92.3 (19.2)	71.7 (7.5)	116.9(11.7)	47.8(11.9)	86.7 (24.4)	153.8 (30.3)	95.6 (51.6)

Caption: *SD* Standard deviation, *DBP* Diastolic arterial pressure, *SBP* Systolic arterial pressure, *LDL-c* Low-density lipoprotein cholesterol, *HDL-c *high density lipoprotein cholesterol, *Wilcoxon rank Sum test, ** Student t test, *** Kruskal Wallis test, **** one-way ANOVA

We evaluated 1,268 individuals; 50.3% were men, 87.1% were 18 years old, 95.3% had none to 11 years of schooling, 55.3% were mixed-raced, 49.6% belonged to socioeconomic class C, 84.1% did not work, 59.2% did not consume alcohol, 96.9% did not smoke, 80.7% had adequate BMI, 11.1% have depression (Table 1).

Table 1 also shows the difference in the means of the outcomes considering the sociodemographic and lifestyle variables. Men had a higher mean glycemia than women. Those with higher schooling had higher mean diastolic blood pressure than their peers. Men and those aged 18 years had higher mean systolic blood pressure when compared to 19 years of age and women, respectively. Adolescents aged 19 years, women, black, and those with adequate nutritional status had higher means of HDL. Women and obese had higher LDL averages. Adolescents aged 19 years, women, obese, and those belonging to the 2^nd^ quartile of PA had higher total cholesterol means. Concerning triglycerides, the highest averages were found among men aged 19 years or older, smokers and obese. Individuals with a sleep duration less than 6 h had higher mean SBP, while those that sleep more than 6 h had a higher mean LDL.

The data in Table 2 show the results of the crude and adjusted analyses concerning the sleep duration associated with the outcomes. The crude analysis showed an association between sleep duration and systolic blood pressure and LDL. Adolescents who have < 6 h of sleep per day have 2.90 mmHg more systolic blood pressure when compared to those who have ≥ 6 h of sleep. Those with < 6 h of sleep per day have 3.33 mg/dL less LDL-c when compared to those with ≥ 6 h of sleep. However, after adjustments to sociodemographic and lifestyle variables, the associations did not remain. That is, there was no association between sleep duration and cardiometabolic risk outcomes.

**Table 2:** Crude and adjusted analysis of the association between sleep duration and cardiometabolic risk factors in adolescents. São Luís, Maranhão, Brazil, 2016/2017

**Variables**	**Crude analysis**		**Adjusted analysis**	
	**β (95%IC)**	***p***	**β (95%IC)**	***p***
**Glycemia (mg/dL)**				
** Sleep duration**		0.407		0.760
≥ 6 h	0		0^a^	
< 6 h	0.82(-1.11;2.75)		0.33(-1.80;2.47)	
**SBP (mmHg)**				
** Sleep duration**		0.001		0.910
≥ 6 h	0		0^b^	
< 6 h	2.90(1.43;4.37)		-0.08(-1.50;1.34)	
**DBP (mmHg)**				
** Sleep duration**		0.202		0.898
≥ 6 h	0		0^b^	
< 6 h	0.58(-0.31;1.48)		0.06(-0.92;1.05)	
**HDL-c (mg/dL)**				
** Sleep duration**		0.145		0.286
≥ 6 h	0		0^a^	
< 6 h	-1.04(-2.44;0.36)		0.93(-0.78;2.63)	
**LDL-c (mg/dL)**				
** Sleep duration**		0.036		0.303
≥ 6 h	0		0^a^	
< 6 h	-3.33(-6.45;-0.22)		-1.90(-5.51;1.71)	
**Total cholesterol (mg/dL)**				
** Sleep duration**		0.098		0.941
≥ 6 h	0		0^a^	
< 6 h	-3.18(-6.94;0.59)		-0.16(-4.55;4.22)	
**Triglycerides (mg/dL)**				
** Sleep duration**		0.308		0.969
≥ 6 h	0		0^a^	
< 6 h	2.98(-2.75;8.71)		0.13(-6.36;6.62)	

*95%IC* 95% confidence interval; *DBP *Diastolic blood pressure; *SBP *systolic blood pressure; *LDL-c *low-density lipoprotein cholesterol; *HDL-c *high density lipoprotein cholesterol. ^a^Model 1 = Adjusted for age, gender, adolescent years of study, skin color, economic class, current work, alcohol consumption, smoking, physical activity, screen time, food consumption; ^b^Model 2 = Adjusted Model 1 + depression

## Discussion

We aimed to analyze the association between sleep duration and cardiometabolic factors in adolescents of the birth cohort (1997/98) in São Luís, Maranhão. In this study, we observed no associations between sleep duration and cardiometabolic factors in adolescents.

Like this study, some studies that used accelerometry in the evaluation of sleep duration also did not observe significant associations with cardiometabolic outcomes in adolescents. The study by Sung et al. [30] with obese American adolescents (10 to 16 years) showed no association between sleep duration and metabolic outcomes. Concerning lipid profile, the presence of heteroscedasticity in the data limited the analysis of the associations. Hjorth et al. [31], reported negative associations between sleep duration and metabolic syndrome scores in Danish children and adolescents aged 8 to 11 years. However, the associations did not remain in the analyses adjusted for gender, age, pubertal stage, leisure physical activity, sedentary behavior, and fat mass index. With the inclusion of the fat mass index in the adjustment model, the authors suggest adiposity could mediate this relationship.

On the other hand, other studies that have also used accelerometers have reported significant associations between sleep duration and blood pressure in adolescents. Javaheri et al. [32] reported a negative association between sleep duration (each one-hour increase) and SBP in American adolescents aged 13 to 16 years and no association for DBP. In the study by Meininger et al., [33] American adolescents aged 11 to 16 years showed lower SBP with each one-hour increase in night sleep, and lower SBP and DBP for each additional hour of daytime sleep. Another study conducted with American adolescents aged 14 to 19 years showed an association between the shorter sleep duration and lower SBP and DBP in the 24 h throughout the 2 days of outpatient monitoring [34].

Although our study showed no association between sleep duration and cardiometabolic factors, studies have suggested some mechanisms that could explain this relationship, such as circadian misalignment, increased sympathetic nervous system activity, and renal sodium retention. Hyperinsulinemia is believed to raise blood pressure by increasing renal sodium retention or stimulating the sympathetic nervous system or a combination of both, which could change cardiometabolic factors [11].

According to the systematic review of observational studies (including questionnaires, self-report of parents and accelerometry) conducted by Quist et al. [11], we observed some evidence of association with cardiometabolic outcomes in children and adolescents. Regarding glycemia (*n *= 21 studies) and blood pressure (*n* = 30 studies), the evidence was more conclusive, whereas regarding the associations with serum lipids (*n* = 14 studies) it was inconsistent. However, the authors [11] point out that it should be considered that most studies relating sleep with published cardiometabolic outcomes were cross-sectional in nature, and subjective measures for sleep assessment often evaluated sleep.

The difference between sleep duration measured by accelerometer and by other methods is an important point. From the total minutes without movement in the wrist in a given period of time [23, 35], accelerometer provides objective information about daily variability and sleep duration, recording information in the home sleep environment, not being influenced by patient expectations and memory bias [35]. However, although lack of body movement is an important indicator of the presence of sleep, accelerometer does not allow the evaluation of subjective aspects of sleep, such as the quality or satisfaction with sleep, [35, 36] and the results are limited to sleep architecture. In this sense, the lack of association may be due to the sleep indicator used in this study (accelerometer), and the results should be interpreted with caution. According to evidence in the literature, the evaluation of sleep by subjective measures may be associated with cardiometabolic outcomes [11], thus, further studies investigating aspects of sleep and these outcomes in adolescence are needed.

The study has some limitations. One of them refers to collecting food consumption data using FFQ, whose quantification is often inaccurate and subjected to memory bias [37]. However, these food consumption data were obtained through a validated instrument for the study population. Another limitation may be related to the losses of participants who used the accelerometer, which may lead to selection bias. It is the adolescents with better health who may have attended to evaluate cardiometabolic factors, which may underestimate the values. In addition, we have the limitation that we cannot distinguish fasting versus non-fasting blood samples.

On the other hand, this study also has strengths. The main strength was the extensive training of the team of interviewers, performed to reduce the biases during data collection. Another strong point was the construction and use of DAG to identify confounding factors, which indicated the minimum set of variables for adjustment, seeking to avoid inadequate or unnecessary adjustments.

It is important to note that the study cannot have the results extrapolated to the population because the sample was not probabilistic.

## Conclusions

These results allow us to observe no association between sleep duration and cardiometabolic risk factors in adolescence. However, these associations may not have occurred at this stage yet and may arise in other life phases. Despite the inexistence of association of factors in this phase, monitoring sleep duration and cardiometabolic risk profile is important to prevent damage in the next phases of the life.

## Supplementary Information

**Additional file 1.** Legend: Sistema de Informações sobre Nascidos Vivos (SINASC)/ Information System on Live Births (SINASC)

## Data Availability

The data that support the findings of this study are available from e-mail rosangela.flb@ufma.br, but restrictions apply to the availability of these data, which were used under license for the current study, and so are not publicly available. Data are however available from the authors upon reasonable request and with permission of Rosangela Fernandes Lucena Batista.

## Abbreviations

SBP: Systolic blood pressure
DBP: Diastolic blood pressure
LDL-c: Low-density lipoprotein cholesterol
HDL-c: High-density lipoprotein cholesterol
DAG: Directed Acyclic Graph
mg/dl: Milligrams per deciliter
mmHg: Millimeters of mercury
NSF: National Sleep Foundation
FFQ: Food frequency questionnaire
Classificação Econômica Brasil – CEB: Economic Classification Brazil
BMI: Body mass index
DSM IV: Diagnostic and Statistical Manual of Mental Disorders
MINI: Mini International Neuropsychiatric Interview
UFMA: Federal University of Maranhão
MA: Maranhão
DECIT: Department of Science and Technology
SD: Standard deviation
95%IC: 95% Confidence interval
