# Supplementary figures and images for: Association between sleep duration and cardiometabolic factors in adolescents
Source: BMC Public Health. 2022 Apr 8;22:686. doi: 10.1186/s12889-022-13119-7 (PMC8991664; doi:10.1186/s12889-022-13119-7)

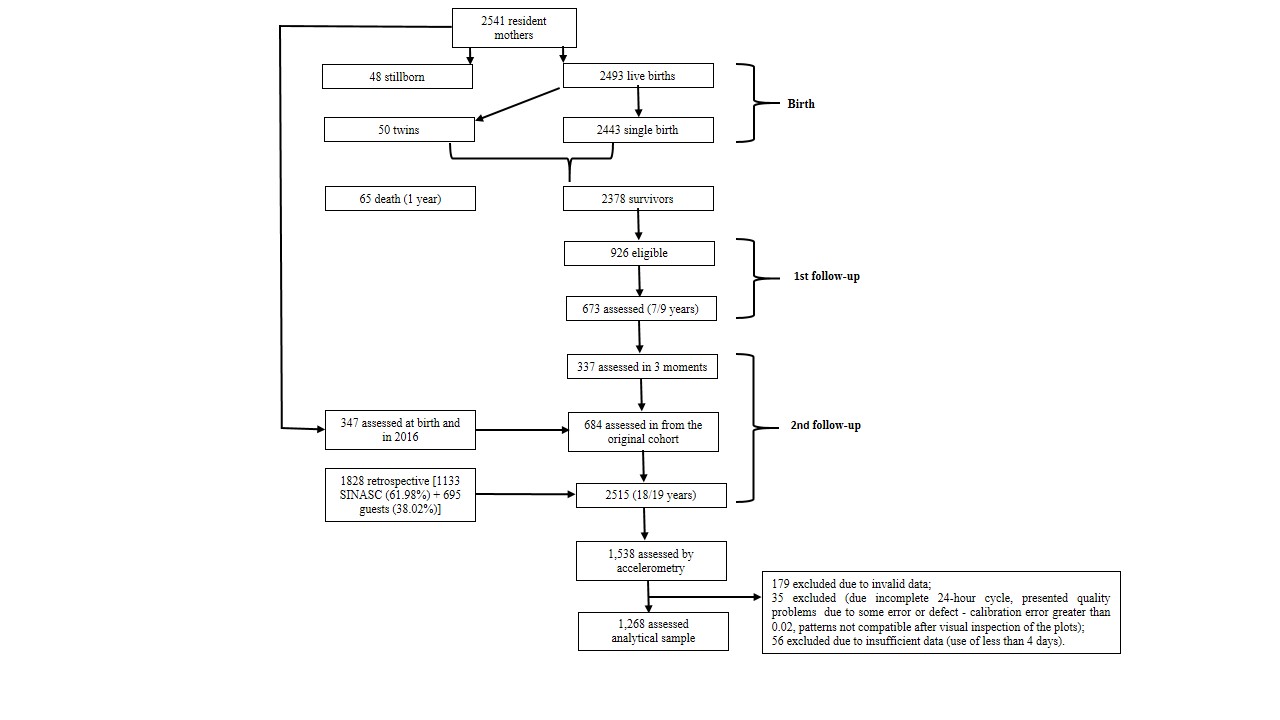

Supplement: Supplementary file 1 — Additional file 1. Legend: Sistema de Informações sobre Nascidos Vivos (SINASC)/ Information System on Live Births (SINASC) [file 12889_2022_13119_MOESM1_ESM.jpg]
